# Clinical Relevance of Interferon Regulatory Family-4 (IRF4) Expression in Newly Diagnosed Patients with Multiple Myeloma

**DOI:** 10.1007/s12288-023-01628-3

**Published:** 2023-01-16

**Authors:** May E. Abdelmonem, Hend A. Nooh, Mona S. El Ashry

**Affiliations:** 1 Faculty of Medicine, Cairo University, Cairo, Egypt; 2 Clinical Pathology Department, National Cancer Institute, Cairo University, Kasr Al Eini Street, Fom El Khalig, P.O Box 11796, Cairo, Egypt

**Keywords:** IRF4, Multiple myeloma, Immuno-histochemistry

## Abstract

Multiple myeloma (MM) is a malignant plasma cell neoplasm with complex biology and heterogenous course. Interferon regulatory factor 4 (IRF4) transcription factor, important key developmental stages of hematopoiesis, represents an excellent potential therapeutic target. The present work aimed to investigate the expression status of IRF4 in the diagnostic bone marrow biopsy (BMB) cores of MM patients. This prospective study included 62 newly diagnosed MM patients. The expression of IRF4 was assessed in the BMB by immunohistochemistry (IHC). The data were correlated to the patients’ clinico-pathological features, response to treatment and survival rates. IRF4 expression was observed in 50% of MM patients (31/62). IRF-4 positive patients were more frequently male patients (*P* = 0.018), have immunoglobulin heavy chain (IgH) translocations (*P* = 0.05) and tended to present with a higher platelets count (*P* = 0.07). Multiple myeloma patients presenting with urine M-protein had worse overall survival (OS) than negative cases (*P* = 0.012). Normocellular BM aspirate (BMA) was associated with better OS than hypercellular and hypocellular BMA (*P* = 0.006). Patchy distribution of plasma cells in BMB was associated with better disease-free survival (DFS) while diffuse infiltration had the worst (*P* = 0.019). Of note, after treatment, MM patients had significantly lower percentage of BMA plasma cells, platelet count, *β2* microglobulin and creatinine levels (*P* = 0.037, < 0.001, 0.022 and 0.026, respectively). Had higher albumin level (*P* = 0.007), compared to initial investigations. No significant association was found between IRF4 expression and the patients’clinical outcomes. Patterns of plasma cells distribution in BMB, BMA cellularity and urine M-protein are prognostically relevant in MM.

**Supplementary Information:**

The online version contains supplementary material available at 10.1007/s12288-023-01628-3.

## Introduction

Multiple myeloma (MM) is an incurable hematologic cancer characterized by the abnormal proliferation of clonal BM plasma cells, with a 5-year OS of approximately 55%, despite the revolutionary novel therapies in the last decade [1, 2]. It is characterized by the secretion of a monoclonal immunoglobulins, affecting multiple organs like renal affection, bony osteolytic lesions and BM infiltration with plasma cells [3, 4].

Multiple myeloma, accounting for 1% of all cancers and 10–15% of all hematologic malignancies, [5, 6], also it is considered to be the second most frequent blood malignancy globally after lymphomas, causing 20–25% of deaths from hematological malignancies [6, 7]. Owing to the trend of global population aging, there was an 126% increase in the incidence of MM worldwide in recent years [8, 9].

At the molecular level, Myeloma is not accounted to be a single disease but a heterogenous disease with numerous subtypes defined by specific gene expression profiles and recurrent chromosomal translocations [10–12]. Many chromosomal abnormalities are frequently presented in myeloma patients like hyperdiploidy and IgH rearrangements [13]. Fluorescent in-situ hybridization (FISH) studies of the BM revealed that about near half of MM patients are characterized by the presence of trisomies in the neoplastic plasma cells (trisomic MM), most of the rest have a translocation involving the IgH locus on chromosome 14q32 (IgH translocated MM), and a small part of patients have both trisomies and IgH translocations [14]. Secondary cytogenetic abnormalities may arise along the disease course, including gain (1q), del(1p), del(17p), del (13), RAS mutations, and MYC translocations [15]. Importantly, the prognostic impact of cytogenetic abnormalities on survival and treatment response in MM vary depending on the disease phase in which they are detected [16, 17].

In pursuit for better prognostication with an better development of risk-adapted treatment, the current IMWG staging system included tumor burden and high-risk cytogenetics, in a new staging system known as (Revised International Staging System R-ISS) [18, 19].

Transcription factor interferon regulatory factor 4 (IRF4), which is located at the 6p25.3 locus, is an important factor of lymphocyte development, differentiation into Ig-secreting plasma cells [20, 21], in addition to its major role in cell proliferation, apoptosis, oncogenic conversion susceptibility and T cell immune reaction [22].

Faulty regulation of IRF4 expression is associated with multiple lymphoid malignancies including MM [21, 23], where in a minority of cases, chromosomal translocation t(6;14) (p25;q32) brings the IRF4 gene under the control of IgH regulatory regions [24, 25].

Experimentally, it was found that the loss of function of IRF-4 gene have resulted in addiction of MM cells “to this abnormal gene expression program. Reduced IRF4 expression causes rapid and extended non-apoptotic cell death, irrespective of genetic etiology [26]. It had been suggested that high expression of IRF4 in MM was associated with worse outcome and inferior survival [27].

In the present study, we investigated the characterization of IRF4 positive cases in newly diagnosed MM patients with special emphasis on the clinical features associated with those cases and their impact on the outcome of the patients.

## Patients and Methods

### Patients

The present study included 62 newly diagnosed MM patients who were diagnosed during the period from May 2017 to December 2019. All patients were presented to the Outpatient Clinic of the Medical Oncology Department, National Cancer Institute (NCI), Cairo University, Egypt. Patients who received any previous chemotherapy outside NCI or with incomplete data were excluded from the study. All patients provided informed consent. The study was performed following the Declaration of Helsinki and was approved by the internal review board of the NCI.

### Methods

The diagnosis of MM was based on the morphological examination of the peripheral blood (PB), BMA and BMB smears, serum protein electrophoresis (SPEP), serum immunofixation (IF), serum lactate dehydrogenase (LDH), albumin (ALB), serum calcium, serum ß2 microglobulin (*β2M*), serum creatinine (sCr), in addition to conventional cytogenetics, FISH. Radiographs (X-ray or CT chest) and magnetic resonance imaging (MRI).

The diagnosis of MM required 10% or more clonal plasma cells in the BM (and/or a biopsy proven plasmacytoma) plus any one or more myeloma defining events (MDE): end-organ damage (hypercalcemia, renal insufficiency, anemia, or bone lesions) attributable to the underlying plasma-cell disorder, BM clonal plasma cells ≥ 60%, serum involved to uninvolved free light chain (FLC) ratio ≥ 100 (provided involved FLC level is ≥ 100 mg/L), or more than 1 focal lesion (≥ 5 mm) on mMRI [5]. The treatment responses were evaluated according to the International Myeloma Working Group Panel Consensus.

#### Immuno-histochemistry

Immunohistochemistry analysis was performed to evaluate the expression of IFR4. Specimens of pre-treatment BM trephine biopsy were transported and fixed in formalin, decalcified in Formic acid-Sodium Citrate and processed to paraffin-wax embedding. Representative sections (3–4 μm) of each case were cut. For each case, 3 slides were prepared: one for Haematoxylin and Eosin (H&E) staining, the second for IHC staining using anti-CD138 and the third IHC assessment of IRF4 using anti-IRF4 (rabbit monoclonal, IgG, Chongqing Biospes Genemed, Biotechnologies, INC), according to manufacturer’s instructions.

Two senior hematopathologists independently scored the percentage of plasma cells and IRF-4 positive cells, using the staining of the endothelium as positive internal control in the tested slides by IRF4, and graded the immunostaining intensity as follows: plain: 0 point; shallow yellow: 1 point; pale yellow: 2 point; sepia: 3 point. For the scoring of positive results, a cut off of 10% was used. Based on the percentage of IRF4 positive cells, the patients were bichromatized into positive (≥ $10\%)$, and negative (< 10%).

Based on staining intensity and the percentage of cells staining positively, the proportion of cells expressing IRF4 were further sub-classified as follows: 0, < 10% of plasma cells or no staining intensity, 1+, $\ge \hspace{0.17em}$10% with light staining, 3+, $\ge \hspace{0.17em}$60% with strong staining intensity, and 2+: for cases not scored as 1+ nor 3+.

#### Cytogenetic Analysis

For conventional cytogenetic analysis, BM specimens were cultured for 24 and 72 h without mitogens, and stained by G-banding, following the standard techniques. In most of the cases, at least 20 metaphases were analyzed using an IKAROS imaging system (Metasystems, Altlussheim, Germany).

Interphase FISH (iFISH) analysis was performed using IgH Breakapart probe (Metasystems, Altlussheim, Germany), according to the manufacturer’s instructions. A minimum of 200 interphase nuclei and 10 metaphases were analyzed using a fluorescence microscope (AxioImager.Z1 mot, Carl Zeiss Ltd., Hertfordshir, UK) with ISIS imaging system (Metasystems, Altlussheim, Germany). The karyotypes were interpreted using the International System for Human Cytogenetic Nomenclature (ISCN 2016) [28].

#### Management of the Patients

After diagnosis of MM, patients were divided into two main groups, transplant eligible and transplant ineligible. Transplant eligible patients with no marked co-morbidities and good performance status (PS 0–2) were offered bortezomib based regimen, namely VCD protocol (Cyclophosphamide 300 mg/m^2^, Bortezomib 1.5 mg/m2, Dexamethasone 40 mg on D1,8,15,22). Patients were requested to repeat SPEP (with IF), s*β2M* monthly and BMA after 12 weeks therapy. Monitor of renal and liver functions were requested before each cycle. Responders continued same regimen and were prepared for Autologous BMT.

Patients with poorer PS & ineligible for transplant were offered either VCD or Lenalidomide. Patients with refractory/progressive disease were offered 2nd line therapy versus best supportive care (BSC) according to their performance and comorbidities. Second line protocols included Lenalidomide/Dexamethasone, VAD and Melphalan and prednisone. All patients were offered supportive treatment including Zoledronic acid and palliative radiotherapy (if clinically/radiologically indicated).

#### Statistical Analysis

Data management and analysis were performed using SPSS, version 22 (IBM, Armonk, Ny, USA). Qualitative data were presented as numbers and percentages, while the quantitative data were presented as median and interquartile ranges (IQR) or mean and standard deviation (SD), according to the appropriate normality test. The comparison between groups were performed using Chi-square test and/or Fisher exact test which appropriate. Mann–Whitney test was used for comparing numerical variables between two groups.

Survival analysis was done using the Kaplan–Meier test, and comparison between survival curves was done using the log-rank test. All tests of hypotheses had been conducted at the alpha level of 0.05, with a 95% confidence interval. All tests were two-tailed. The OS was calculated from the date of diagnosis till the date of last follow-up or death due to any cause while the DFS was calculated from the date of the diagnosis until the date of relapse, death during induction, death during CR, or second malignant neoplasms.

## Results

### Patients Characteristics

Among the 62 patients with MM, 39 (62.9%) patients were male while 23 patients (37.1%) were female, with a median age of 55 years at diagnosis. By skeletal survey, all the patients presented with bony lesions, of which 50/62 (80.6%) patients had single or multiple osteolytic lesions while 12/62 patients (19.4%) presented with pathological fractures. Out of 62 patients, 22 (35.5%) presented with soft tissue plasma cells. Baseline characteristics are shown in Table 1.

**Table 1:** Patient characteristics at diagnosis

Parameter	Frequency (%)	Parameter	Frequency (%)
Age*	55 (42–85)	sCalcium (mg/dl)	
< 65	50 (80.6)	> 12	14 (22.6)
≥ 65	12 (19.4)	< 12	48 (77.4)
Gender		LDH (U/L)	
Male	39 (62.9)	> 270	22 (35.5)
Female	23 (37.1)	< 270	40 (64.5)
TLC (× 10^9^)		ISS	
< 4	6 (9.7)	I	24 (38.7)
≥ 4	56 (90.3)	II	17 (27.4)
Hb (gm/dL)		III	21 (33.9)
< 8	8 (12.9)	BMA plasma cells %*	27 (2–84)
≥ 8	54 (87.1)	< 10	6 (9.7)
Platelets count × 10^9^		10–59	28 (45.2)
< 150	12 (19.4)	≥ 60	28 (45.2)
≥ 150	50 (80.6)	BMB CD138 + *	30 (11.0–95)
Urine M protein		10–59%	39 (62.9)
No	54 (87.1)	≥ 60%	23 (37.1)
Yes	8 (12.9)	Cytogenetics Results	
IF heavy chain		IgH-translocations	19 (30.6)
IgG	58 (93.5)	Hyperdiploid	14 (22.6)
Non-IgG	4 (6.5)	Hypodiploid	8 (12.9)
IF light chain		Combined IgH and trisomies	5 (8)
Kappa	49 (79.0)	Dup(1q)	7 (11.3)
Lambda	13 (21.0)	MCN*	46 (41–94)
ALB (g/dl)		Patients’ response to therapy	
< 3.5	28 (45.2)	CR	41(66.1)
≥ 3.5	34 (54.8)	Refractory	12 (19.4)
sCr (mg/dL)		Early death	8 (12.9)
≥ 1.768	18 (29.0)	Relapse	
< 1.768	44 (71.0)	Yes	15 (24.2)
*sβ2M (mg/L)*		No	26 (41.9)
< 3.5	25 (40.3)	Death	
3.5–5.5	16 (38.0)	Yes	24 (38.7)
≥ 5.5	21 (33.9)	No	38 (61.3)

Data are shown as a number (percentage) or a *median (interquartile range)

*ALB* albumin, *BMA* bone marrow aspirate, *BMB* bone marrow biopsy, *CR* complete remission, *Hb* hemoglobin, *IF* immunofixation, *ISS* international staging system, *LDH* lactate dehydrogenase, *sβ2M* serum β2microglobulin, *sCr* serum creatinine, *TLC* total leucocytic count

### Baseline Laboratory Characteristics

Different percentages of plasma cells were infiltrating the BMA with a mean (± SD) of 29.1% (± 23.2). Upon BMB examination, 39/62 patients (62.9%) presenting with hypercellular marrow, 20/62 (32.3%) had normocellular marrow and 3/63 (4.8%) had hypocellular marrow. Diffuse infiltration by plasma cells was the most frequent pattern in BMB being shown in 28/62 patients (45.2%), followed by interstitial infiltration in 26/62 (41.9%) while patchy distribution was found in 8/62 (12.9%), with a median (range) of CD138 + plasma cells of 30% (11.0–95). Thirty-four patients had BMB fibrosis; 20 (32.3%) patients had stage I, 11 (17.7%) had stage II and 3 (4.8%) had stage III.

M Protein was found in the serum of all patients, while it was found in the urine of 8/62 (12.9%) of patients. Monoclonal M-band was found in 60/62 (96.7%) wile biclonal bands were present in 2/62 (3.3%) patients.

A total of 26 patients (41.9%) had abnormal karyotyping of which 7 (11.3%) patients had dup (1q), and 19 (30.6%) patients had IgH rearrangement. Combined cytogenetic analyses revealed that 38/62 (61.3%) patients had cytogenetic abnormalities of which combined trisomies and IgH translocations were found in 5/62 (8%) of cases. Other baseline laboratory findings are illustrated in Table 1.

### Response to Treatment

Fourteen patients (22.6%) received Bortezomib while 7 (11.3%) patients undergone BMT. Forty-one of 62 cases (66.1%) achieved CR after which 15 (36.6%) relapsed while 8 cases (12.9%) died during induction. Comparison between the baseline and follow up laboratory investigations are summarized in Table 2.

**Table 2:** Relation of initial and follow up laboratory investigations

Laboratory investigations	Initial		Follow up		*P* value
	Mean ± SD	Median (IQR)	Mean ± SD	Median (IQR)	
Hb (g/dL)	10.2 ± 2.1	9.9 (5.7–16.2)	10.6 ± 2.4	10.8 (5.4–15.5)	0.059
TLC (× 10^9^/L)	6.9 ± 2.5	6.4 (2.3–15.8)	12.7 ± 46.5	6.0 (2–360)	0.829
Plt (× 10^9^/L)	239.2 ± 97.8	253 (17–451)	215.1 ± 88.2	226 (8.5–463)	**< 0.001**
Calcium (mg/dl)	9.3 ± 1.4	36.5 (13–142)	8.7 ± 0.8	8.8 (5.6–10.3)	0.525
ALB (g/dl)	3.38 ± 0.86	3.61 (1.10–5.10)	3.7 ± 1.7	3.5 (1.1–15.1)	**0.007**
*sβ2M (mg/L)*	5.46 ± 3.81	3.95 (1–21)	4.2 ± 3.7	3.5 (1.5–24)	**0.022**
Urea (mg/dl)	42.8 ± 22.6	36.5 (13–142)	44 ± 30.6	35 (19–218)	**0.385**
sCr (mg/dL)	1.38 ± 1.42	0.9 (0.50–7.80)	1.3 ± 1.2	1 (0–7.3)	**0.026**
LDH (u/l)	253.7 ± 84.5	247 (100–536)	254.8 ± 51.6	260 (145–350)	0.797
Total protein (g/dl)	8.56 ± 1.97	8.08 (4.40–15.40)	7 ± 1.4	7.0 (2.3–13.3)	0.584
BMA plasma cells %	29.1 (± 23.2)	27 (2–84)	9 (± 12.4)	4 (1–60)	**0.037**

Bold signifies the results with statistical significance

After treatment, although 13/54 (24%) patients had BMA plasma cells ≥ 10%, there was significant decrease in the percentage of BMA plasma cells (*P* = 0.037) positive BMB infiltration was present in 35/54 (64.8%). There was a significant decrease in the Plt count, s*β2M* and sCr levels (*P*≤ 0.001, 0.022 and 0.026, respectively). In addition, there was a significant increase in the levels of ALB (*P* = 0.007) and a trend of increase in the Hb level (*P* = 0.059). M band was found in 26/62 (41.9%) patients. By skeletal survey after treatment, regressive response was found in 18/54 (33.3%), progressive in 5/54 (9.3%) while 31/54 (57.4%) patients were of stationary response.

### Association of IRF4 with MM Patients’ Characteristics

**Fig. 1:**
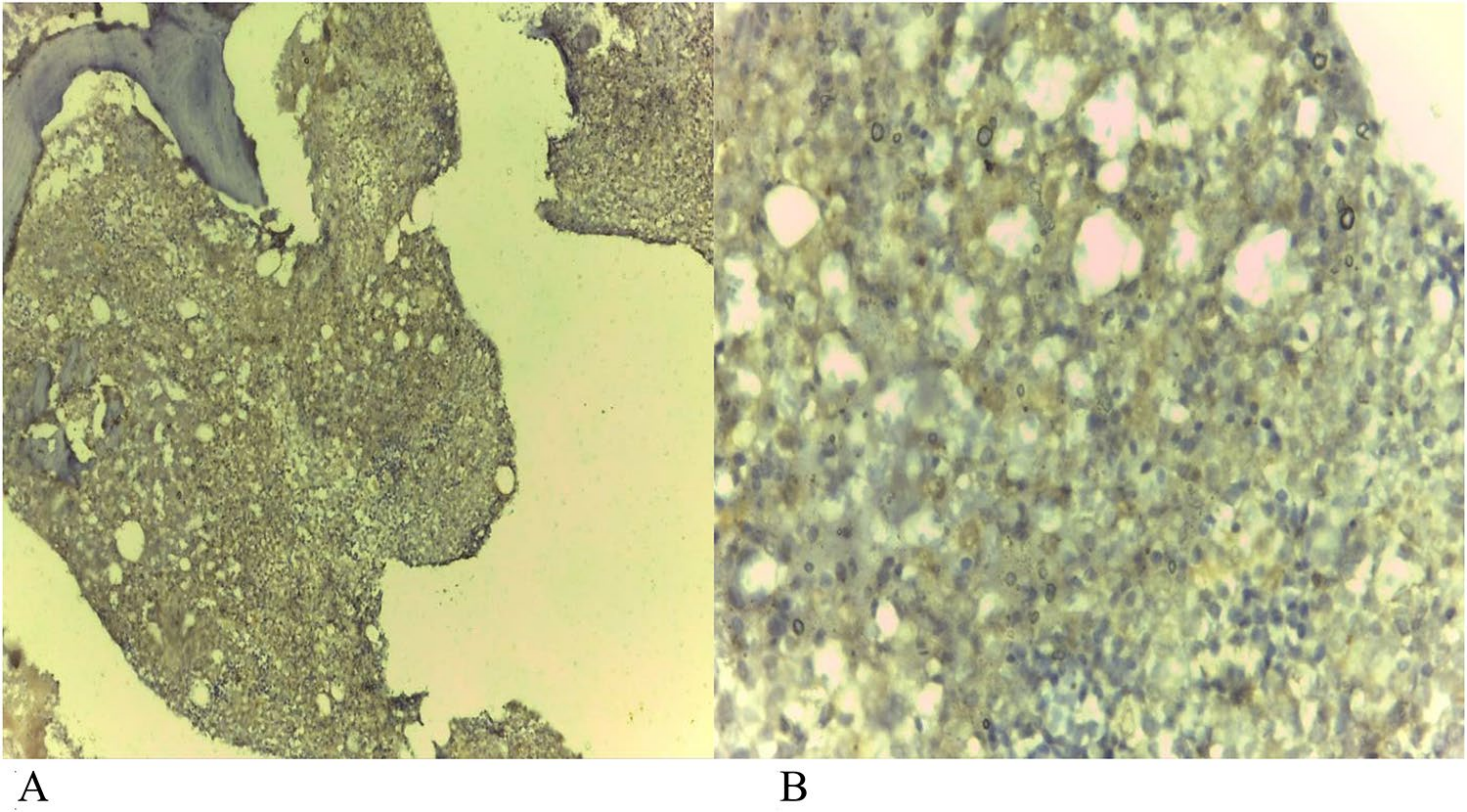
**A** 10 × magnification, **B** 40 × magnification Leica Microscope, BMB IHC with IRF4 positive Myeloma cells

By IHC, there was positive expression of IRF4 in 31/62 (50%) patients. as shown in Fig. 1. Upon scoring of IRF-4 expression, depending on the intensity and distribution, 19/62 (30.6%) were classified as +, 5/62 (8.1%) as ++ and 7/62 (11.3%) as +++. The relations between IRF4 staining with scoring system and different laboratory parameters are shown in the supplementary table S1.

IRF-4 was expressed in plasma cells nuclei and not observed in neutrophils, lymphocytes nor macrophages. Out of 31 IRF-4 positive cases, 24 were males compared to 7 patients were females (61.5% vs. 30.4%, *P* = 0.018). There was a trend of IRF4-positive cases to have higher platelet count than negative cases with a *P value of* 0.081. In the same context, there was a non-significant trend of patients with 1+ to have higher platelet count than 2+ and 3+ (*P* = 0.07).

**Fig. 2:**
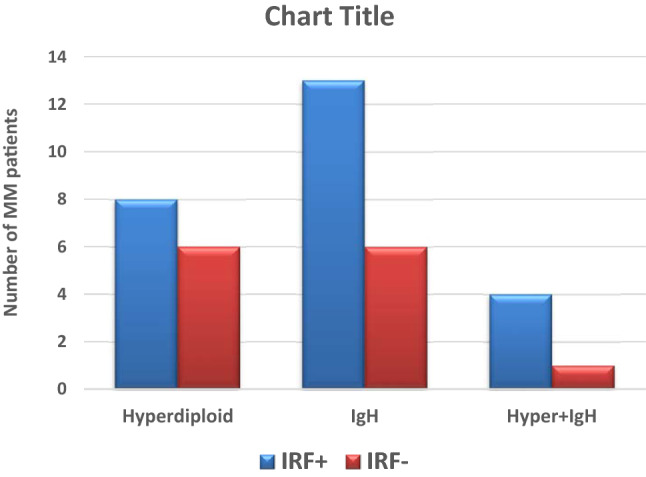
Cytogenetic presentations in myeloma patients in relation to IRF4 expression

Interestingly, rearrangements of IgH were more prevalent in IRF4-positive cases than negative cases (68.4% vs. 31.6%) with a *P* value of 0.054. No other significant relation between IRF4 expression and patient characteristics as illustrated in Table 3 and Fig. 2.

**Table 3:** Association of IRF4 with initial laboratory investigations

Patients’ characteristics	IRF-4 expression		*P v*alue
	IRF4-positive (N = 31)	IRF4-negative (N = 31)	
Age	52.9 (± 10.7)	58.1 (± 11.8)	0.258
TLC	7.08 (± 1.80)	6.71(± 3.10)	0.471
Hb	10.26 (± 2.40)	10.11 (± 1.81)	0.662
PLT	236 (± 104)	243 (± 92)	0.772
ALB (g/dl)	3.31 (± 0.80)	3.44 (± 0.92)	0.641
*sβ2M (mg/L)*	5.01(± 3.19)	5.91 (± 4.35)	0.508
sCr	1.4 (± 1.4)	1.4 (± 1.5)	0.568
Urea	41.8 (± 22.1)	43.7 (± 23.4)	0.356
Calcium	9.2 (± 1.2)	9.4 (± 1.6)	0.577
LDH	255.4 (± 92.4)	252.1 (± 77.2)	0.901
Total Protein	8.83 (± 2.32)	8.29 (± 1.54)	0.836
BMA plasma cells %	32.4 (± 24.2)	25.9 (± 22)	0.321
MCN	48.48 (± 8.85)	46.65 (± 3.07)	0.104
Sex			***0.018***
Male	24 (61.5%)	15 (38.5%)	
Female	7 (30.4%0	16 (69.6%)	
P.Fracture			0.520
No	26 (52.0%)	24 (48.0%)	
Yes	5 (41.7%)	7 (58.3%)	
Urine.M.ptn			1.000
Positive	4 (50.0%)	4 (50.0%)	
Negative	27 (50.0%)	27 (50.0%)	
BMA.Cellualrity			1.000
Normo	14 (50.0%)	14 (50.0%)	
Hypo	3 (50.0%)	3 (50.0%)	
Hyper	14 (50.0%)	14 (50.0%)	
BMB.Cellularity			1.000
Normo	10 (50.0%)	10 (50.0%)	
Hypo	2 (66.7%)	1 (33.3%)	
Hyper	19 (48.7%)	20 (51.3%)	
BMB.infiltration			*0.937*
Diffuse	13 (46.4%)	15 (53.6%)	
Interstitial	14 (53.8%)	12 (46.2%)	
Patchy	4 (50.0%)	4 (50.0%)	
BMB.Fibrosis			*1.00*
No	14 (50.0%)	14 (50.0%)	
Yes	17 (50.0%)	17 (50.0%)	
Cytogenetic Analyses			*0.712*
Normal	10 (47.6%)	11 (52.4%)	
Abnormal	20 (52.6%)	18 (47.4%)	
Karyotyping			*0.675*
Diploid	20 (50.0%)	20 (50.0%)	
Hypodiploid	3 (37.5%)	5 (62.5%)	
Hyperdiploid	8 (57.1%)	6 (42.9%)	
IgH-translocations			***0.054***
Positive	13 (41.9%)	6 (19.4%)	
Negative	18 (58.1%)	25 (80.6%	
ISS			
I	13	11	
II	9	8	
III	9	12	
Patients’ response to therapy			0.421
CR	Yes	22 (71%)	
Refractory	6 (19.4%)	6 (19.4%)	*1.00*
Early Death	3 (9.7%)	5 (16.1%)	0.707
Relapse			
No	11(52.4%)	12 (70.6%)	0.242
Yes	10 (43.6%)	5 (29.4%)	

Bold signifies the results with statistical significance

Italic indicates non statistically significant results

### Impact of IRF4 and Other Prognostic Factors on the Clinical Outcome of MM Patients

**Fig. 3:**
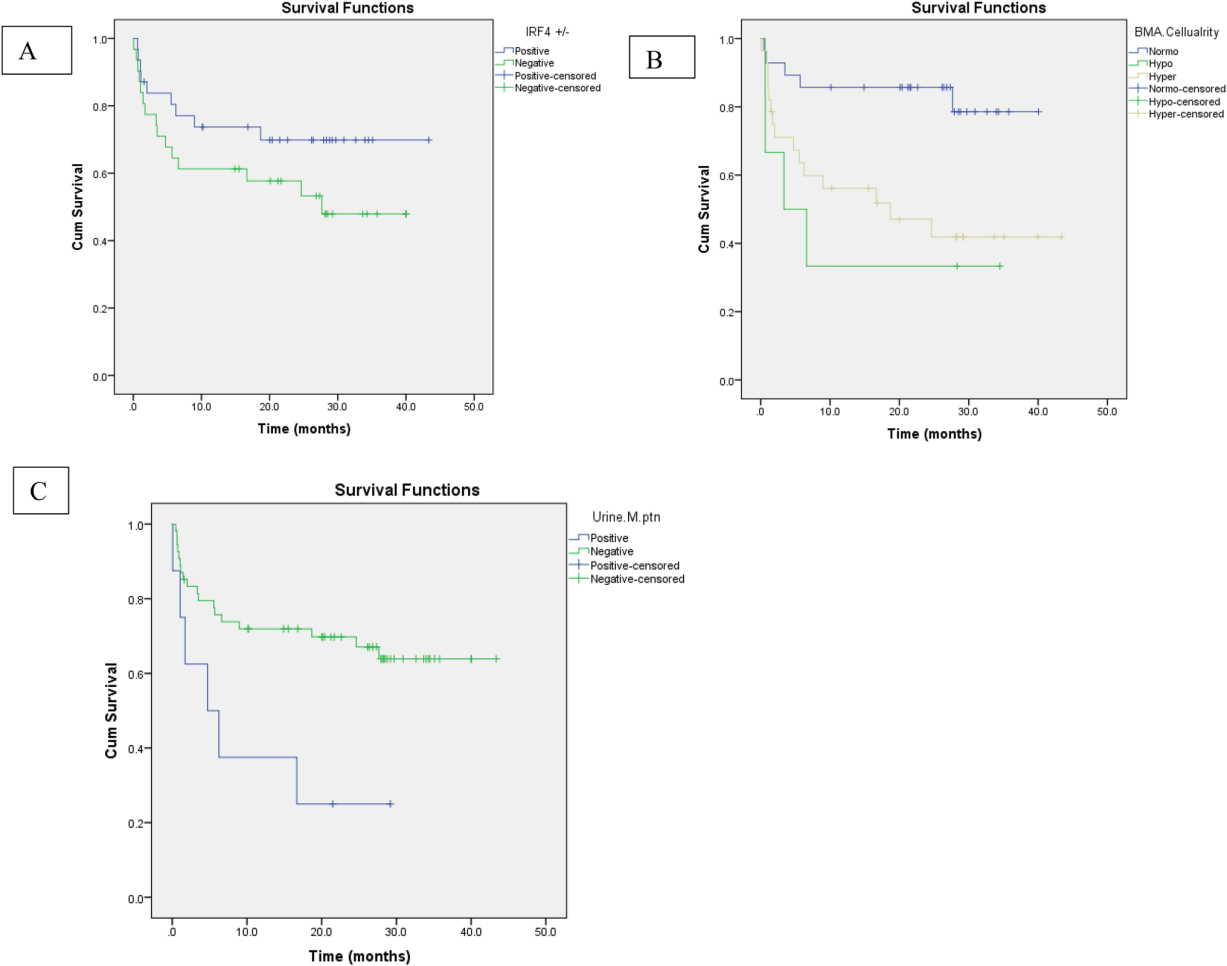
Overall survival of **A** IRF±, **B** urine M-protein and **C** BMA cellularity

The median follow up of OS was 20.8 months with a range of 0.1–43.4 months and a mean (± SD) of 18.5 (± 13.1) months. The known prognostic factors had been investigated and revealed improved OS for MM patients with higher Hb level at diagnosis (*P* = *0.047*), and non-significant better survival among patients with lower calcium level (*P* = *0.07*)*.* In addition, patients with Urine M-protein had worse OS than negative cases (2 years-cumulative survival % of 25% compared to 66.7% of negative cases) with a *P* value of 0.012. Normocellular BMA was associated with better survival than hypercellular and hypocellular marrow (*P* = 0.006) as illustrated in Fig. 3.

**Fig. 4:**
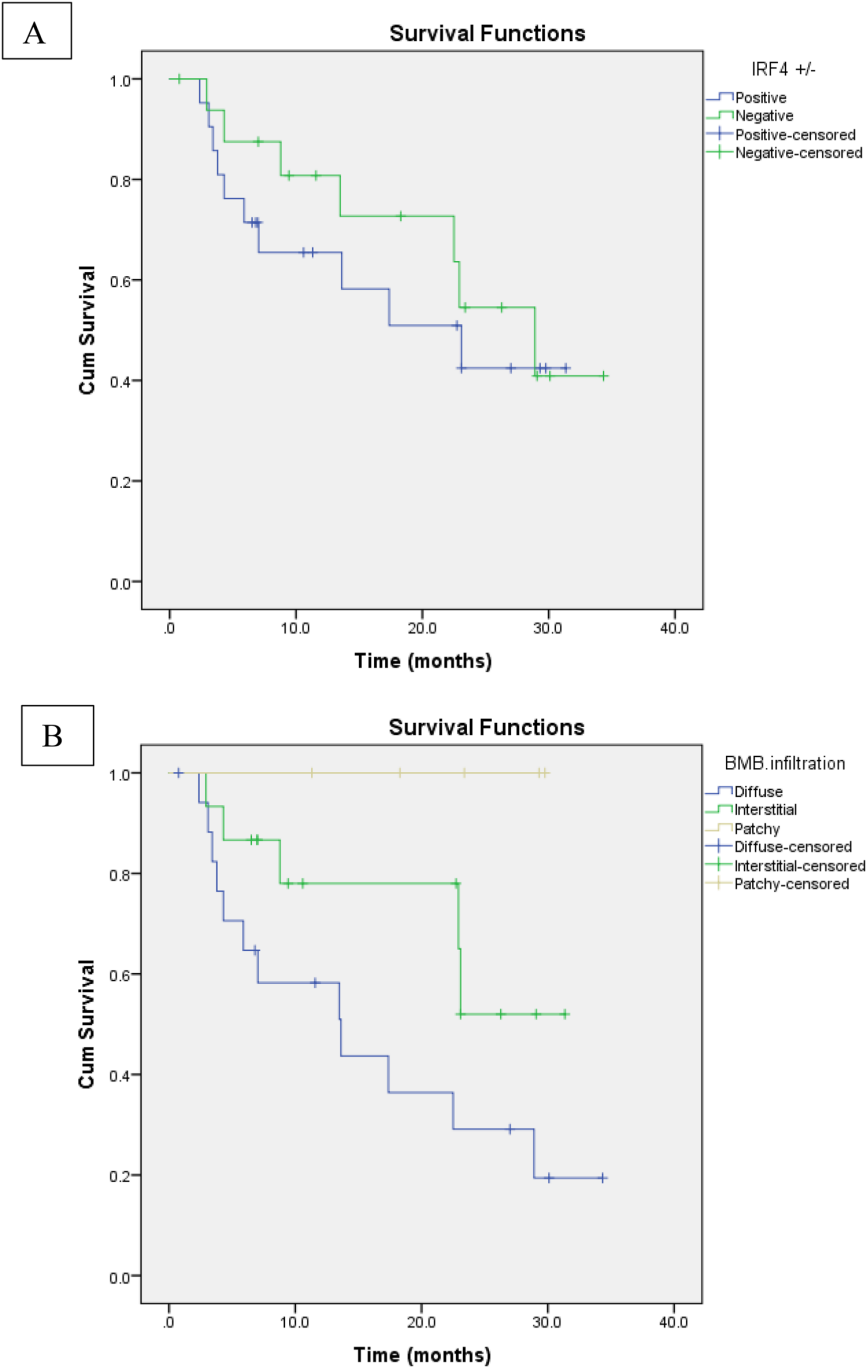
Disease free survival of **A** IRF±, **B** BMB pattern of infiltration

After a median (range) follow up period of 12.5 (0.8–34.3) months and a mean (± SD) of 15.4 (± 10.4) months, all prognostic factors had no significant relation with DFS except for the pattern of BMB infiltration as patchy distribution was associated with better DFS while diffuse infiltration had the worst survival (*P* = *0.019*) as shown in Fig. 4. No significant association between IRF4 expression and DFS nor OS. Although the median OS in ISS stage I was longer than that of stage II and stage III (23.18 vs 19.1 and 11.12 months, respectively), the relation failed to show statistical significance (0.266). Relations between different prognostic factors with OS and DFS are illustrated in Table 4.

**Table 4:** Relation between prognostic factors with Disease free survival overall survival of 2 years

	Overall survival				Disease free survival			
	n	Events	Cumulative survival (%)	*P* value	n	Events	Cumulative survival (%)	*P* value
Whole group	62	24	61.3		38	17	55.3	
Sex								
Male	39	12	69.2	0.152	25	10	60.0	0.560
Female	23	12	47.8		13	7	46.2	
M component								
One band	57	22	61.4	0.865	35	17	51.4	0.212
2 bands	5	2	60.0		3	0	100.0	
IF.Light.Chain								
Kappa	49	17	65.3	0.283	30	12	60.0	0.311
Lambda	13	7	46.2		8	5	37.5	
Urine.M.ptn								
Positive	8	6	25.0	***0.012***	21	6	71.4	N/A
Negative	54	18	66.7		2	2	0.0	
BMA.Cellualrity								
Normo	28	5	82.1	***0.006***	15	9	40.0	N/A
Hypo	6	4	33.3		12	3	12	
Hyper	28	15	46.4		1	0	1	
BMB.infiltration								
Diffuse	28	14	50.0	0.318	5	0	100.0	***0.019***
Interstitial	26	7	73.1		18	8	55.6	
Patchy	8	3	62.5		20	9	55.0	
BMB.Fibrosis								
No	28	11	60.7	0.855	16	8	50.0	0.846
Yes	34	13	61.8		16	7	56.3	
Conv.Cyto								
Normal	28	8	71.4	0.155	12	6	50.0	0.999
Abnormal	26	13	50.0		25	10	60.0	
Cytogenetics								
Normal	21	5	76.2	0.245	24	12	50.0	0.587
Abnormal	21	5	55.3		4	1	75.0	
Karyotyping								
Diploid	40	14	65.0	0.253	10	4	60.0	0.701
Hypodiploid	8	5	37.5		13	4	69.2	
Hyperdiploid	14	5	64.3		16	7	56.3	
IRF4±								
Positive	23	9	60.9	0.616	38	17	55.3	0.585
Negative	29	14	51.7		25	10	60.0	

Bold signifies the results with statistical significance

italic indicates non statistically significant results

## Discussion

Multiple myeloma (MM) is an incurable clonal plasma cell malignancy characterized by multiple remissions and relapses. Despite the improvement of MM treatment by the use of proteasome inhibitors and immunomodulatory agents, it remains an incurable disease as almost all the patients who survive initial treatment will eventually relapse, requiring further therapy [29]. Given that MM cells depend on IRF4 for survival [30, 31], deletion of IRF4 gene results in apoptosis of plasma cells as reported by Low et al. [32]. Blocking IRF4 expression or interfering with its transcriptional network provide an attractive and applicable therapeutic option for the many subtypes of MM [26].

Compared to other cohorts [19, 31, 33–37], our patients were younger (median of 55 years vs. 65 years), presented more commonly with osteolytic lesions (90% vs 35%) and had more frequently higher levels of LDH (35% vs. 12%). In line with other series [33], combined abnormal cytogenetics analyses were detected in 61.3% of our patients and 22.6% had hyperdiploidy. The frequency of IgH rearrangements was lower than that reported by other cohorts [36, 37], this discrepancy could be explained by the fact that FISH was performed on unsorted plasma cells in our study.

Our frequency of IRF4 positivity of 50% was comparable to that of a previous study reporting IRF4 positivity in 65% of MM patients, as assessed by IHC [22]. IRF-4 expression was more frequently found in male patients. Other than that, no association was found between IRF-4 expression and the clinical parameters, such as age, pathological fractures, sCr, ALB, and LDH, in agreement with Bai et al. Consistent with other reports, IRF4 expression was positively associated with IgH rearrangements. This is consistent by the fact that IRF4 overexpression may result of activating mutations or recurrent translocations involving the 6p25 *IRF4* locus, of which IgH is one of the potential partner genes in patients with t(6;14) (p25;q32) [24, 25].

Previous studies [22, 38] suggested that the high expression of IRF4 is correlated with poor survival and dismal outcome. However, our current study did not support this conclusion as we didn’t find a statistical association between IRF4 expression and patients’ OS nor DFS. This could be explained by the small number of patients in our study in comparison to others or the presence of underlying genetic or factors related to patients’ clinical characteristics that necessitates more comprehensive study on larger cohort.

The known prognostic factors had been investigated and revealed improved OS for MM patients with higher Hb level at diagnosis*,* and non-significant better survival among patients with lower calcium level, in line with other studies. In the same context, MM patients presented with urine M-protein had an inferior OS compared to patients lacking urine M-protein*,* previous study comparing the progression of MGUS and smoldering myeloma with severe myeloma regarding urinary proteins [39].

Of note, there was a statistical association between pattern of infiltration of plasma cells in the biopsy sections and DFS, where patchy infiltration was associated with longer DFS compared to interstitial and diffuse infiltration, which could be explained by the lesser load of plasma cells in the patchy pattern of infiltration, this finding was in agreement with Rajkumar et al. [40].

We found in our study like others [41], that plasma cell loads in the BM was not statistically associated to patients survival**.** However, this finding was in contrast to Wei et al. who considered that the initial high plasma cells found in the marrow was a remarkable predictor of patients ability to relapse [42]. In the same context, we noted a statistical significant decline of plasma cell burden after treatment, followed by positive infiltration of BM which we could explain this observation like Tandon et al. who found increased sensitivity of myeloma patients to treatment but with decreased long term response to those treatments and reduced outcomes [43]. Although about two thirds of our patients cohort achieved CR after first line chemotherapy, no significant correlation with improved OS or DFS could be found, which was in contrast to others reporting there was an ultimate association between patients achieving CR from the first line chemotherapy and improved OS and DFS [44–46].

In conclusion, MM is a mix of different disease entities. Hemoglobulin levels, patterns of plasma cells distribution in BMB, BMA cellularity and urine M-protein are prognostically relevant in MM. No significant association was found between IRF4 expression and survival rates of the patients.

### Supplementary Information

Below is the link to the electronic supplementary material.

Supplementary file1 (DOCX 17 kb)
