# Supplementary material for: Clinical Relevance of Interferon Regulatory Family-4 (IRF4) Expression in Newly Diagnosed Patients with Multiple Myeloma
Source: Indian J Hematol Blood Transfus. 2023 Jan 16;39(4):525–36. doi: 10.1007/s12288-023-01628-3 (PMC10542031; doi:10.1007/s12288-023-01628-3)
Supplement: Supplementary file 1 — Supplementary file1 (DOCX 17 kb) [file 12288_2023_1628_MOESM1_ESM.docx]

**Supplementary Tables:**

**Table S1: Association of IRF4-scores with laboratory investigations**

| **Parameter** | **IRF4 final score** | | | | **P value** |
| --- | --- | --- | --- | --- | --- |
|  | **Negative (N=31)** | **+ (N=19)** | **++ (N=5)** | **+++ (N=7)** |  |
| **Age** | 58.13 (±11.81) | 54.21 (±9.32) | 49.20 (±17.64) | 51.86 (±9.48) | 0.258 |
| **TLC** | 6.71 (±3.10) | 7.21 (±1.94) | 6.76(±1.83) | 6.95 (±1.61) | 0.471 |
| **Hb** | 10.11 (±1.81) | 10.59 (±2.14) | 10.26(±3.68) | 9.36 (±2.15) | 0.662 |
| **Plt** | 242.68 (±92.48) | 251.26 (±92.73) | 197.8(±114.43) | 220.29 (±132.41) | 0.772 |
| ***sβ2M (mg/L)*** | 5.91 (±4.35) | 4.8 (±3.48) | 21.8 (±19.7) | 36 (±30.2) | 0.306 |
| **Urea** | 43.74 (±23.44) | 36.58 (±18.69) | 47.6 (±28.09) | 51.86 (±25.31) | 0.356 |
| **sCr** | 1.39 (±1.49) | 1.49 (±1.73) | 1.03 (±0.48) | 1.27 (±0.51) | 0.568 |
| **Calcium** | 9.35 (±1.58) | 9.32 (±0.91) | 9.22 (±1.49) | 8.89 (±1.89) | 0.577 |
| **LDH** | 252.1 (±77.21) | 247.37 (±81.58) | 235.00 (±91.24) | 291.57 (±122.82) | 0.901 |
| **ALB** | 3.44 (±0.92) | 3.46 (±0.65) | 3.4 0(±0.60) | 2.83 (±1.16) | 0.641 |
| **Total Protein** | 8.29 (±1.54) | 9.00 (±2.39) | 8.16 (±2.54) | 8.84 (±2.21) | 0.836 |
| **BMA plasma cells%** | 25.9 (±22) | 33.8 (±23.5) | 21.8 (±19.7) | 36 (±30.2) | 0.650 |
| **MCN** | 46.65 (±3.07) | 49.89 (±10.97) | 48.0(±3.08) | 45.00 (±1.73) | 0.104 |
| **Sex** |  |  |  |  |  |
| Male | 15 (38.5%) | 14 (35.9%) | 4 (10.3%) | 6 (15.4%) | o.138 |
| Female | 16 (69.6%) | 5 (21.7%) | 1 (4.3%) | 1(4.3%) |  |
| **P.Fracture** |  |  |  |  |  |
| No | 24 (48.0%) | 15 (30.0%) | 4 (8.0%) | 7 (14.0%) | 0.676 |
| Yes | 7 (58.3%) | 4 (33.3%) | 1 (8.3%) | 0 (0.0%) |  |
| **Urine.M.ptn** |  |  |  |  |  |
| Positive | 4 (50.0%) | 2 (25.0%) | 1 (12.5%) | 1 (12.5%) | No P value |
| Negative | 27 (50.0%) | 17 (31.5%) | 4 (7.4%) | 6 (11.1%) |  |
| **Serum.IG** |  |  |  |  |  |
| Normal | 28 (56.0%) | 13 (26.0%) | 3 (6.0%) | 6 (12.0%) |  |
| Decreased | 1 (14.3%) | 4 (57.1%) | 2 (28.6%) | 0 (0.0%) |  |
| Increased | 2 (40.0%) | 2 (40.0%) | 0 (0.0%) | 1 (20.0%) |  |
| **BMA.Cellualrity** |  |  |  |  |  |
| Normo | 14 (50.0%) | 10 (35.7%) | 4 (14.3%) | 0 (0.0%) | No P value |
| Hypo | 3 (50.0%) | 1 (16.7%) | 0 (0.0%) | 2 (33.3%) |  |
| Hyper | 14 (50.0%) | 8 (28.6%) | 1 (3.6%) | 5 (17.9%) |  |
| **BMB.Cellularity** |  |  |  |  |  |
| Normo | 10 (50.0%) | 7 (35.0%) | 2 (10.0%) | 1 (5.0%) | No P value |
| Hypo | 1 (33.3%) | 1 (33.3%) | 0 (0.0%) | 1 (33.3%) |  |
| Hyper | 20 (51.3%) | 11 (28.2%) | 3 (7.7%) | 5 (12.8%) |  |
| **BMB.infiltration** |  |  |  |  |  |
| Diffuse | 15 (53.6%) | 11 (39.3%0 | 0 (0.0%) | 2 (7.1%) | No P value |
| Interstitial | 12 (46.2%) | 6 (23.1%) | 4 (15.4%) | 4 (15.4%) |  |
| Patchy | 4 (50.0%) | 2 (25.0%) | 1 (12.5%) | 1 (12.5%) |  |
| **BMB.Fibrosis** |  |  |  |  |  |
| No | 14 (50.0%) | 10 (35.7%) | 1 (3.6%) | 3 (10.7%) | 0.654 |
| Yes | 17 (50.0%) | 9 (26.5%) | 4 (11.8%) | 4 (11.8%) |  |
| **Cyt.Result** |  |  |  |  |  |
| Normal | 11 (52.4%) | 5 (23.8%) | 2 (9.5%) | 3 (14.3%) | 0.836 |
| Abnormal | 18 (47.4%) | 13 (34.2%) | 3 (7.9%) | 4 (10.5%) |  |
| **Karyotyping** |  |  |  |  |  |
| Diploid | 20 (50.0%) | 12 (30.0%) | 3 (7.5%) | 5 (12.5%) | No P value |
| Hypodiploid | 5 (62.5%) | 1 (12.5%) | 0 (0.0%) | 2 (25.0%) |  |
| Hyperdiploid | 6 (42.9%) | 6 (42.9%) | 2 (14.3%) | 0 (0.0%) |  |
| **IgH** |  |  |  |  |  |
| Positive | 6 (19.4%) | 8 (42.1%) | 2 (40.0%) | 3 (42.9%) | 0.237 |
| Negative | 25 (80.6%) | 11 (57.9%) | 3 (60.0% | 4 (57.1%) |  |
